# Numerical Modeling of Intraventricular Flow during Diastole after Implantation of BMHV

**DOI:** 10.1371/journal.pone.0126315

**Published:** 2015-05-11

**Authors:** Boyang Su, Foad Kabinejadian, Hui Qun Phang, Gideon Praveen Kumar, Fangsen Cui, Sangho Kim, Ru San Tan, Jimmy Kim Fatt Hon, John Carson Allen, Hwa Liang Leo, Liang Zhong

**Affiliations:** 1 National Heart Research Institute of Singapore, National Heart Centre Singapore, Singapore, Singapore; 2 Department of Biomedical Engineering, National University of Singapore, Singapore, Singapore; 3 Department of Surgery, National University of Singapore, Singapore, Singapore; 4 Institute of High Performance Computing, ASTAR, Singapore, Singapore; 5 Duke-NUS Graduate Medical School, Singapore, Singapore; Northwestern Polytechnical University, CHINA

## Abstract

This work presents a numerical simulation of intraventricular flow after the implantation of a bileaflet mechanical heart valve at the mitral position. The left ventricle was simplified conceptually as a truncated prolate spheroid and its motion was prescribed based on that of a healthy subject. The rigid leaflet rotation was driven by the transmitral flow and hence the leaflet dynamics were solved using fluid-structure interaction approach. The simulation results showed that the bileaflet mechanical heart valve at the mitral position behaved similarly to that at the aortic position. Sudden area expansion near the aortic root initiated a clockwise anterior vortex, and the continuous injection of flow through the orifice resulted in further growth of the anterior vortex during diastole, which dominated the intraventricular flow. This flow feature is beneficial to preserving the flow momentum and redirecting the blood flow towards the aortic valve. To the best of our knowledge, this is the first attempt to numerically model intraventricular flow with the mechanical heart valve incorporated at the mitral position using a fluid-structure interaction approach. This study facilitates future patient-specific studies.

## Introduction

Surgical replacement of a dysfunctional valve with a heart valve prosthesis is a viable option for patients suffering from severe heart valve disease. Various prosthetic heart valves have been devised, and bileaflet mechanical heart valves (BMHVs) are widely implanted, owing to long life span and durability [1]. However, the non-physiological flow phenomena (e.g. hinge jet, squeeze flow and regurgitation jet) make BMHVs vulnerable to calcification and thromboebolism [2]. Numerous studies have been carried out to understand the blood flow around BMHV so as to optimize hemodynamic performance and minimize adverse effects. Computational fluid dynamics (CFD) is a widely adopted technique in the study of BMHV, as it has the capability to predict detailed flow in three-dimension (3D). One critical challenge in BMHV simulation is the interaction between leaflets and blood flow. Simplified approaches include prescribing the leaflet motion and simulating flow phenomena at certain instants [3–5]. With the advancement of numerical algorithms, recent numerical simulations use fluid-structure interaction (FSI) to accurately predict leaflet dynamics using either monolithic or partitioned approach [6–8]. The monolithic method solves the governing equations of structure and fluid simultaneously and is computationally demanding. The partitioned approach governs structure and fluid separately and updates information iteratively at the interface until convergence criteria are satisfied. The partitioned approach is more popular, owing to its capability of utilizing sophisticated structure and fluid solvers [6,9–11]. During a numerical modeling, grids have to cope with the large leaflet rotation in a cardiac cycle, and two distinct methods have been developed: fixed grid and moving grid [1]. For the fixed grid method, the entire volume of a numerical model is discretized into fixed grids as a background, and a separate mesh is generated for moving structure, which is immersed in the background grids. Therefore, the remaining volume is in the fluid domain, and the boundary grids are not boundary-conforming. Boundary forces must be introduced into the governing equations to ensure no-slip boundary condition. Although the fixed grid method avoids the difficulty of generating conforming grids and deforming grids, the background grids should have high spatial resolution in the region swept across by the structure in order to accurately predict the interaction [12]. The moving grid method employs arbitrary Lagrangian-Eulerian (ALE) formulation of Navier-Stokes to take into account the grid movement. The velocity of interface node is arbitrary and defined by the Lagrangian motion of the interface between fluid and structure to ensure a body-fitted mesh. However, the inner nodes do not have to move with the local flow speed but vary smoothly and arbitrarily between Lagrangian and Eulerian approaches [13]. Intermediate remeshing is necessary when a large deformation is involved such as rotating heart valve leaflets. For the numerical simulations of mechanical heart valves and heart ventricles, ANSYS FLUENT software is the most widely used CFD solver using ALE approach and the corresponding experimental validations have been conducted using Particle Image Velocimetry (PIV) [6,7,14].

A number of *in-vitro* and *in-vivo* studies have been conducted to study BMHV at mitral position and reported in the literature. Faludi and colleagues found that the intraventricular flow was more complex after the implantation and that based on comparisons using echocardiographic PIV the major vortex rotated in the opposite direction relative to a normal non-implanted subject [15]. Using magnetic resonance imaging (MRI), Macheler and colleagues studied the intraventricular flow patterns induced by different BMHV orientations [16]. Although the resultant intraventricular flow was closely related to the orientation, it was significantly different from the normal subject regardless of orientation. *In-vivo* studies are usually incompetent to quantify the flow parameters, owing to poor spatial and temporal resolutions and subject variations. PIV measurements have been extensively conducted in *in-vitro* studies. Pierrakos and Vlachos investigated the vortex formation after different prosthetic mitral valves in a left heart simulator to quantify the hydraulic efficiencies of BMHV in anatomical and anti-anatomical orientations [17]. Querzoli and colleagues revealed that BMHV preserved most of the beneficial features, compared with tilting valve [18]. However, most of the ventricular measurements are conducted in two-dimension (i.e. cross-sections of complex 3D flow) so that the parameter values may be underestimated. Due in part to the difficulty of modeling the beating left ventricle, the numerical studies of BMHV at the mitral position are quite limited [1].

In this study, a 25 mm BMHV at the mitral position has been numerically simulated with an idealized ventricle downstream. The lower portion of the ventricle was assumed to be a truncated spheroid with its motion prescribed for a normal subject. In order to predict leaflet dynamics accurately, the BMHV leaflets were modeled using FSI approach. For ease of programming, the partitioned approach was adopted and the blood flow was solved using a commercial CFD code, which utilizes ALE method to handle large mesh deformation. Owing to its close connection to mitral valve dynamics, the diastolic phase has been greatly concerned, during which 3D vortical flow evolves [19–25]. The numerically predicted intraventricular flow is comparable to other numerical and experimental results found in the literature.

## Method

**Fig 1:**
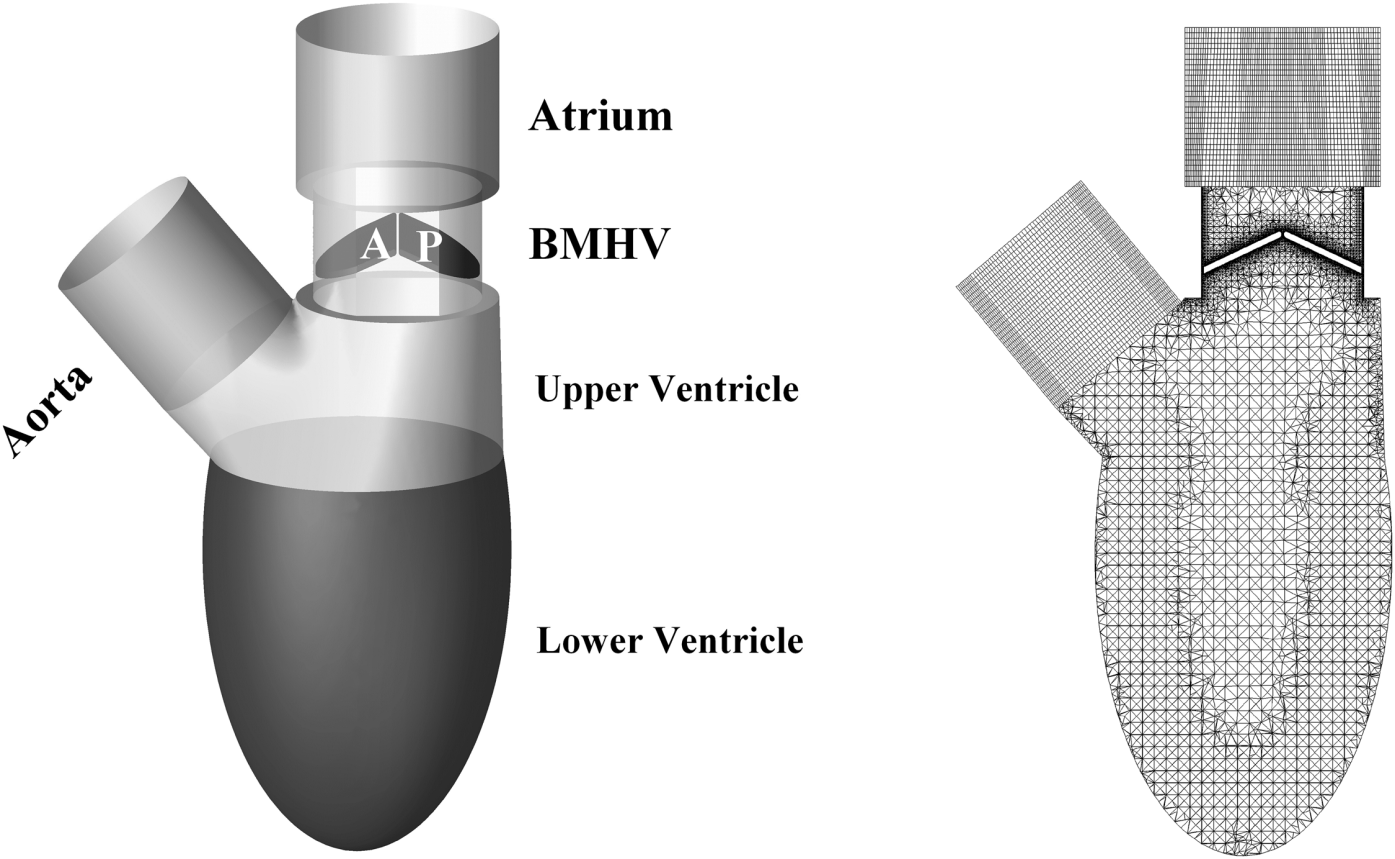
Geometry and mesh of the ideal ventricle. A: anterior leaflet. P: Posterior leaflet.

The geometry of the left ventricle at the beginning of diastole is shown in Fig 1. The lower portion of the ventricle was a truncated spheroid with the long-to-short axis ratio of 2 over the entire cardiac cycle, and its volume at time instant, *t*, is expressed as:

$$V(t)=\pi \cdot \frac{c{\left(t\right)}^{3}}{a^{2}}\left\{\frac{2}{3}+\sqrt{1-\frac{\alpha^{2}\cdot r^{2}}{c{\left(t\right)}^{2}}}+\frac{{\left(-\sqrt{1-\frac{\alpha^{2}\cdot r^{2}}{c{\left(t\right)}^{2}}}\right)}^{3}}{3}\right\}$$ (1)

where *α* is the long-to-short-axis ratio; *c(t)* is the long axis; and *r* is the radius of the intersection between the upper and lower portion of the ventricle (Detailed information is available in [26]. In this study, *r* was 18 mm and *c(t)* varied from 38 to 50 mm during diastole. The corresponding minimum and maximum ventricular volume were 64 and 144 ml, respectively. The resultant ejection fraction was 56%, which is within normal range of a healthy subject. To simplify the numerical analysis, the 25 mm ATS Open Pivot Standard Heart Valve was modeled without a hinge. Both atrium and aorta were represented by straight tubes with diameters of 25 mm and 20.8 mm, respectively. In addition, the aortic valve was excluded in this model, as it have negligible impact on the intraventricular flow during diastole. The angle between the mitral and aortic orifices was 140°.

ANSYS ICEMCFD (Version 14.0) was used to generate grids, which had higher spatial resolution around leaflet as demonstrated in Fig 1 (The 3D mesh model is shown in S1 Text). The selected cross-section was in the middle of the idealized left ventricle, perpendicular to the axis of the leaflet hinge. At fully closed position, the tiny clearance gap between leaflet and housing with respect to the left ventricle model was quite difficult to handle grid generation and deformation. Therefore, leaflets were scaled down, resulting in a clearance gap of 150 μm [6,26,27]. As the leaflet hinge was removed for simplification, the opening angle was numerically constrained between 25° and 85° [28]. Hexahedral grids were generated in atrium and aorta, while the ventricle and valves were meshed with tetrahedral grids to accommodate large mesh deformation induced by the beating ventricle and rotating leaflets. The numerical model included 2.8×10^6^ cells, which consisted of 2.3×10^5^ hexahedral cells and 2.57×10^6^ tetrahedral cells. Spring-based smoothing method alone could not cope with large leaflet motion, therefore a local remeshing algorithm was enabled, which could handle overskewed and/or out-of-length-scale grids.

The internal flow of the numerical model was simulated using the commercial CFD solver ANSYS FLUENT (Version 14.0), which utilizes the finite volume method in ALE formulation of the Navier-Stokes equations to solve the motion of moving ventricle and leaflets. The integral forms of continuity and momentum equations are written as:

$$\frac{\partial }{\partial t}\int_{V}\rho dV+\int_{S}\rho \left(\vec{v}-\vec{v_{b}}\right)\cdot \vec{n}dS=0$$ (2)

$$\frac{\partial }{\partial t}\int_{V}\rho \vec{v}dV+\int_{S}\left(\rho \vec{v}\left(\vec{v}-\vec{v_{b}}\right)+pI-\vec{\tau }\right)\cdot \vec{n}dS=0$$ (3)

where *ρ* is the fluid density; $\vec{v}$ is the velocity vector of fluid; $\vec{v_{b}}$ is the velocity vector of moving boundary; $\vec{n}$ is the outwardly directed vector normal to *dS*; *S* is the boundary of the control volume, *V*; *p* is the pressure; *I* is the unit tensor; and $\vec{\tau }$ is the viscous stress tensor. Blood flow was assumed to be laminar and Newtonian with constant dynamic viscosity of 3.5 mPa∙s and density of 1,050 kg/m^3^ [6,26,27,29].

To model the interaction between blood flow and leaflets, the leaflet motion was solved using FSI method. The BMHV leaflet is rigid in rotation around the hinge, and its motion is governed by:

$$\ddot{\theta }+\xi \dot{\theta }=\frac{M}{I}$$ (4)

where *M* is the torque; *I* is the moment of inertia; and ξ is the damping coefficient. As mentioned in [1], the damping coefficient induced by hinge frictions is small relative to the flow forces experienced by the leaflet, and its experimental value is unavailable. As a result, the damping term is neglected in numerical simulations, and Eq 4 is essentially Newton's second law for rotation. Partitioned approach was adopted in this study so that the equations governing leaflets and blood flow could be coupled at interface using Gauss-Seidel like iteration scheme as expressed in Eq 5.

$${\ddot{\theta }}_{i}^{n+1,k+1}={\ddot{\theta }}_{i}^{n+1,k}+\omega^{n+1,k}\left(\frac{M_{i}^{n+1,k}}{I_{i}}-{\ddot{\theta }}_{i}^{n+1,k}\right)$$ (5)

where *i = 1*,*2* indicates the leaflet; *k* is the number of FSI iteration within one time step; *n* is the number of time step; and ω is the relaxation factor. The Aitken relaxation method was implemented to update the relaxation factor at each time step due to its overall simplicity and computational efficiency [30]. FSI iteration was implemented using Scheme language in conjunction with user-defined functions (UDFs), which were coupled to FLUENT (Detailed information is available in S1 Text).

**Fig 2:**
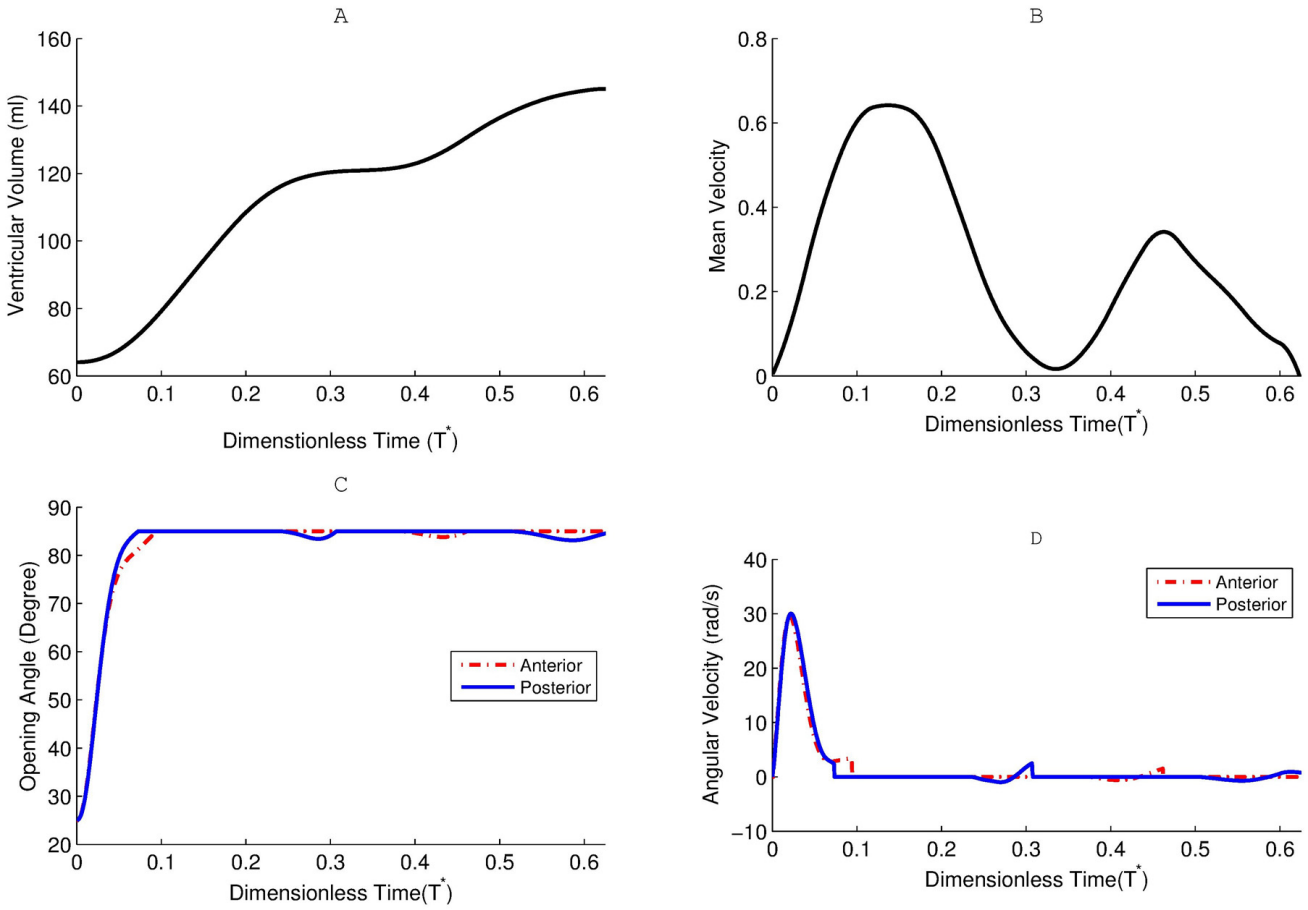
Plots of (a) ventricular volume, (b)mitral flow velocity, (c) leaflet angular position and (d) leaflet angular velocity during diastole. T* = t/T. t: flow time. T: cardiac period. Anterior leaflet: dash-dot line. Posterior leaflet: solid line.

No-slip boundary condition was applied to walls and leaflets. Heart rate was set at 62 beats per minute, corresponding to one complete cardiac cycle of 0.967 s. After performing the time step size dependence test, the optimal computational time step size was determined to be 0.484 ms [9,22,31]. The deformation of ventricular wall was prescribed using UDF based on Eq 1. In a normal subject, the diastolic phase consists of early rapid filling (E-wave), diastasis, and late atrial filling (A-wave) as demonstrated in Fig 2a. As the blood could only flow into the left ventricle through the mitral valve, the temporal derivative of ventricular volume was the flow rate through mitral valve, which was applied to the inlet (Fig 2b). The velocity waveform consists of two crests with peak velocity magnitudes of 0.65 m/s (early diastole) and 0.37 m/s (atrial systole), respectively. A constant static pressure of 110 mm Hg was applied to the outlet. It is worth noting that the static pressure applied at outlet does not affect internal flow field, because only pressure gradient plays a role in Navier-Stokes equations [26].

In this transient simulation, pressure-based solver was selected, and SIMPLEC scheme was adopted for the pressure-velocity coupling. To increase the accuracy, the spatial discretizations of pressure and momentum were of second-order. Due to the limitation of FLUENT, the temporal discretization was of first order when dynamic mesh was enabled. Convergence criteria for all the flow parameters such as continuity, pressure and velocity were set to 10^–5^.

## Results

As demonstrated in Fig 2, the diastole includes three phases: rapid filling, diastasis and atrial systole. The rapid filling starts when the left ventricular pressure falls below the atrial pressure and the pressure gradient opens the mitral valve. The blood flows into the left ventricle rapidly to compensate the ventricular dilation. After achieving a maximal velocity, the ventricle dilates more slowly and the magnitude of atrio-ventricular pressure gradient declines. During the diastasis, the atrio-ventricular pressure gradient is almost zero and the filling is essentially maintained by the momentum of the blood from pulmonary veins in a normal left ventricle. The atrial systole is the contraction of left atrium and ends when the mitral valve closes.

### Leaflet Dynamics

Fig 2c and 2d depict leaflet dynamics during diastole, responding to the beating ventricle. During the rapid filling phase, both leaflets opened rapidly (Fig 2c), owing to the inlet flow acceleration (Fig 2b). In addition, leaflets experienced fast angular acceleration and the peak angular velocity was 30 rad/s, which occurred at T* = 0.02. After that, the angular velocity of leaflet declined dramatically, as the leaflet orientation became more align with the transvalvular flow direction. Although the inlet flow at the atrium was uniform, the leaflets of symmetric BMHV behaved differently from each other, especially when the opening angle was above 70°. The posterior leaflet (solid) firstly reached the maximum opening angle of 85° at the angular velocity of 2.3 rad/s, while the anterior leaflet (dash-dot) was slightly reaccelerated before hit the blocking mechanism at 3.5 rad/s. Both leaflets fully opened before the peak of rapid filling phase, and mostly remained the maximum opening angle during the diastasis and atrial systole.

### Transvalvular and Intraventricular Flow Patterns

**Fig 3:**
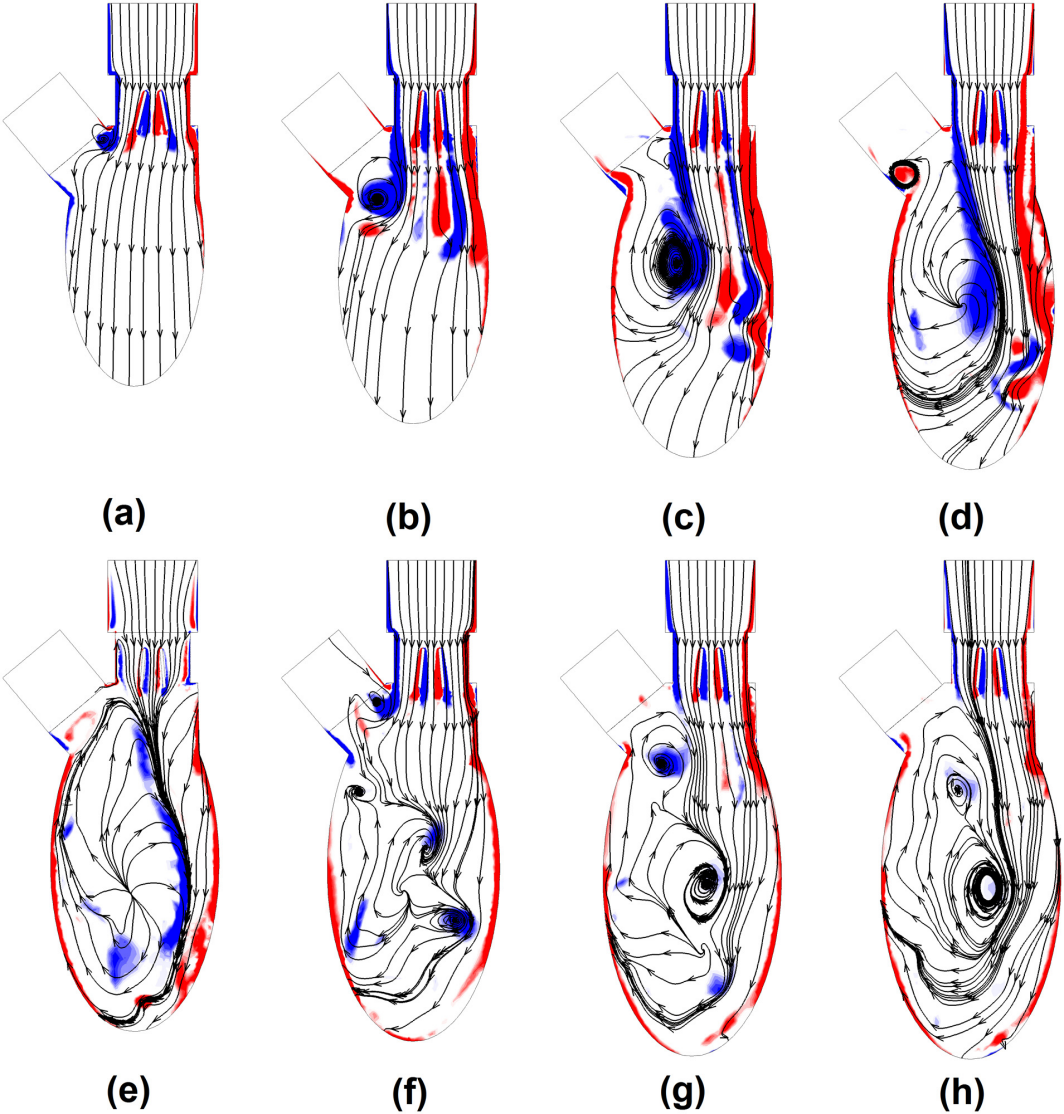
Development of out-of-plane vorticity contours during diastole. (a) T* = 0.05, (b)T* = 0.125, (c)T* = 0.2, (d)T* = 0.25, (e)T* = 0.325, (f)T* = 0.45, (g) T* = 0.55, (h) T* = 0.6. (S1 Video).

Fig 3 illustrates out-of-plane vorticity contours and planar flow streamlines in the middle plane during diastole. At onset of rapid filling, the mitral valve started opening with shear layers developed on both sides of each leaflet in Fig 3a. The sudden cross-sectional area expansion between the valve housing and left ventricle resulted in flow separation and vortices. As the anterior vortex was less constrained, it was relatively large in size. The clockwise rotating vortex slightly entrained the adjacent jet flow and retarded the opening of anterior leaflet (Fig 2c 0.02<T*<0.075. Therefore, the transvalvular flow was asymmetric with the central jet tilting towards anterior vortex. In Fig 3b, continuous injection of jet flow through the orifice drove the vortices downward and the anterior leaflet finally reached the maximum opening angle (Fig 2c). The jet flow further penetrated towards the apex and the region influenced by the anterior vortex increased in Fig 3c. As the anterior vortex started dominating the intraventricular flow, it tilted the transvalvular flow towards posterior wall in Fig 3d, which explained the swing of posterior leaflet (Fig 2c 0.24<T*<0.31). During the diastasis, the anterior vortex moved further away from the leaflets, so its influence declined in Fig 3e. Furthermore, the flow through the mitral orifice was marginal, and the interactions between vortices continued, leading to dissipations of vortices. Despite the opening of BMHV, the flow phenomena during atrial systole were similar to those during rapid filling phase but with weaker strengths. Shear layers developed on leaflets and housing and the newly formed vortex near the root of aorta were observed in Fig 3f. This vortex grew and moved towards the apex in Fig 3g, leading to the closing of the anterior leaflet (Fig 2c 0.44<T*<0.46). Eventually, the vortices induced during the atrial systole merged into clockwise intraventricular flow developed during the rapid filling phase in Fig 3h.

**Fig 4:**
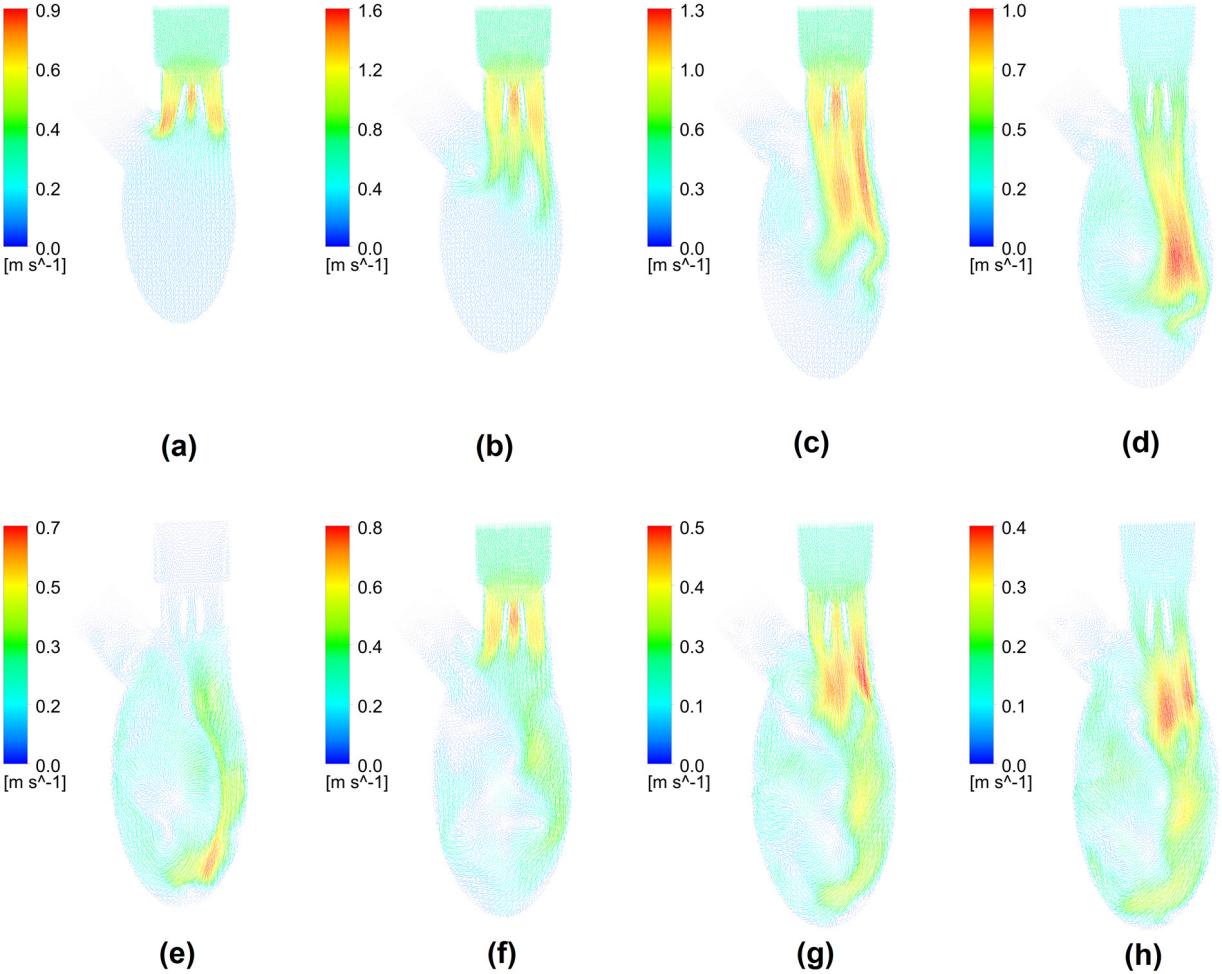
Development of planar velocity during diastole. (a) T* = 0.05, (b)T* = 0.125, (c)T* = 0.2, (d)T* = 0.25, (e)T* = 0.325, (f)T* = 0.45, (g) T* = 0.55, (h) T* = 0.6.

Fig 4 shows planar flow vectors on the middle plane at the same instants selected in Fig 3, and it reveals the development of transvalvular and intraventricular flows on macro scale. The fundamental feature of transvalvular flow was the triple-jet pattern, which consisted of two lateral jets between each leaflet and housing and a central jet between leaflets, and its strength varied with the inlet velocity magnitude. Owing to its physical structure, the flow velocity of central jet between leaflets was slightly higher, compared with lateral jets. During the rapid filling phase, the triple jet propagated towards apex and merged into each other. The jet flow accelerated firstly and the maximum velocity occurred in the central jet with the magnitude of 1.6 m/s. After the peak of rapid filling phase, the head of jet flow moved further downwards and its strength declined gradually. The conspicuous anterior vortex that was initiated near the aortic root played an import role in directing the intraventricular flow, which was developed from flow separation. Its clockwise rotating direction slightly tilted the triple-jet flow, which eventually impinged on the curved posterior wall. The curvature of ventricular wall further promoted the clockwise rotating anterior vortex. During diastasis, the jet flow during the rapid filling phase reached the apex and the its strength further decreased with the maximum velocity of 0.7 m/s. Similarly, a weaker triple-jet flow and an anterior vortex were observed during the atrial systole. The jet flow also moved along the posterior wall and merged into the clockwise intraventricular flow initiated during the rapid filling phase.

### Pressure Gradient Patterns

**Fig 5:**
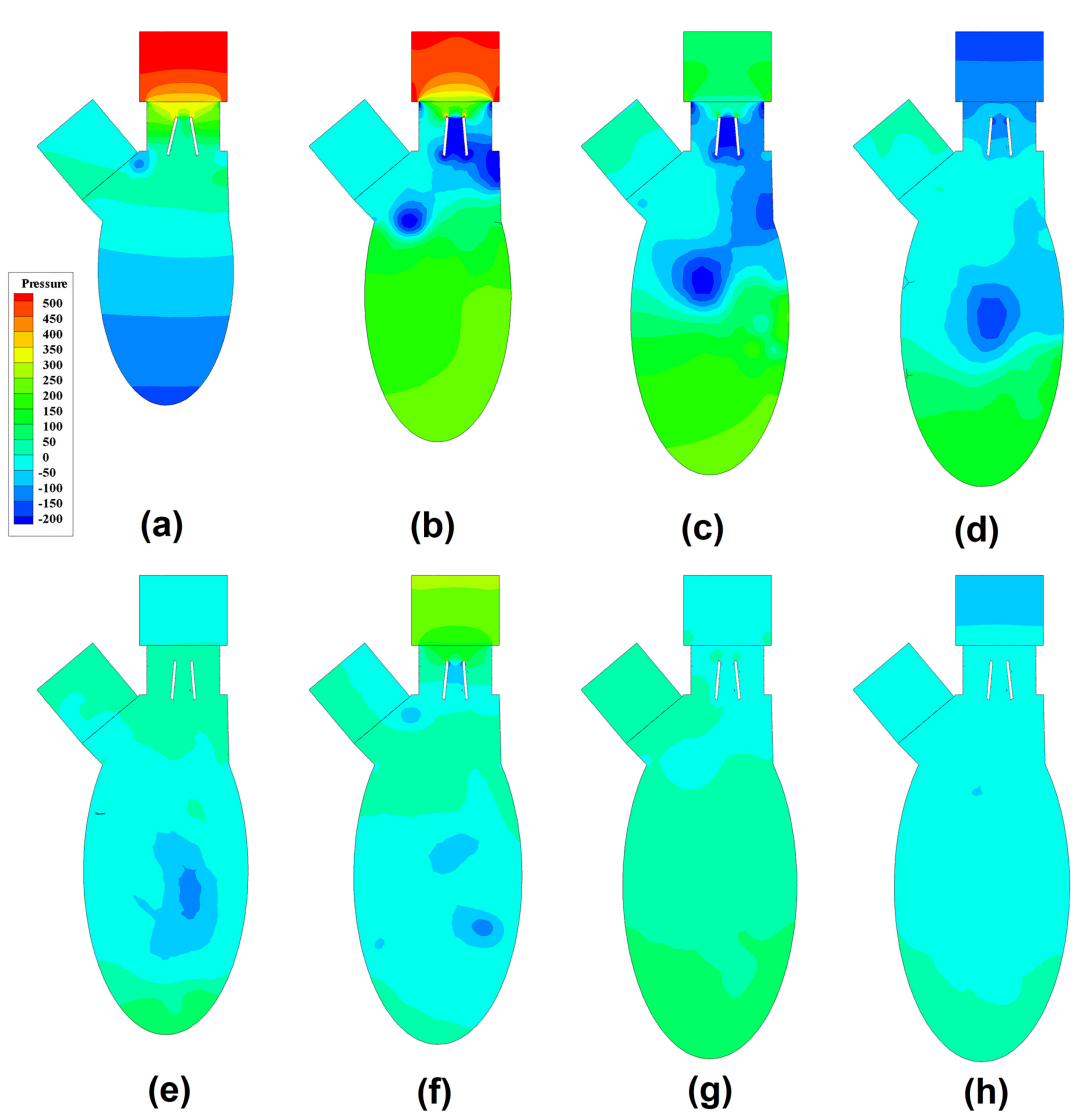
Development of pressure gradient pattern during diastole. (a) T* = 0.05, (b)T* = 0.125, (c)T* = 0.2, (d)T* = 0.25, (e)T* = 0.325, (f)T* = 0.45, (g) T* = 0.55, (h) T* = 0.6.

In order to demonstrate the pressure gradient, the static pressure at the outlet was set as the reference, and these patterns at the corresponding instants are shown in Fig 5. Near the onset of rapid filling phase, the pressure gradient along vertical direction drove the forward flow through the mitral orifice in Fig 5a and 5b. Around the root of aorta, a low pressure region appeared, which was the core region of anterior vortex (Fig 3b). This low pressure region entrained the anterior leaflet and explained the delay of its opening (Fig 2c 0.02<T*<0.075). After the peak of inlet flow, the pressure gradient across the mitral valve declined in Fig 5c and 5d. As the low pressure region penetrated further downwards in Fig 5b–5d, its influence over the leaflet diminished and the pressure gradient between the core (dark blue) and the surrounding region declined (light blue). During the diastasis as shown in Fig 5f, the location of the low pressure region moved further towards the apex and the pressure gradient became more uniform, which corresponded to the mixing of vortices in Fig 3. At the early stage of atrial systole, a low pressure region was generated in Fig 5f, which was the core region of newly formed vortex (Fig 3f). As the jet flow during the atrial systole was weaker, the low pressure region penetrated towards the apex slowly and the corresponding pressure gradient related to the surrounding region was lower, compared with the observations in rapid filling phase. Consequently, the newly formed low pressure region diminished during the rest of the atrial systole in Fig 5g and 5h.

**Fig 6:**
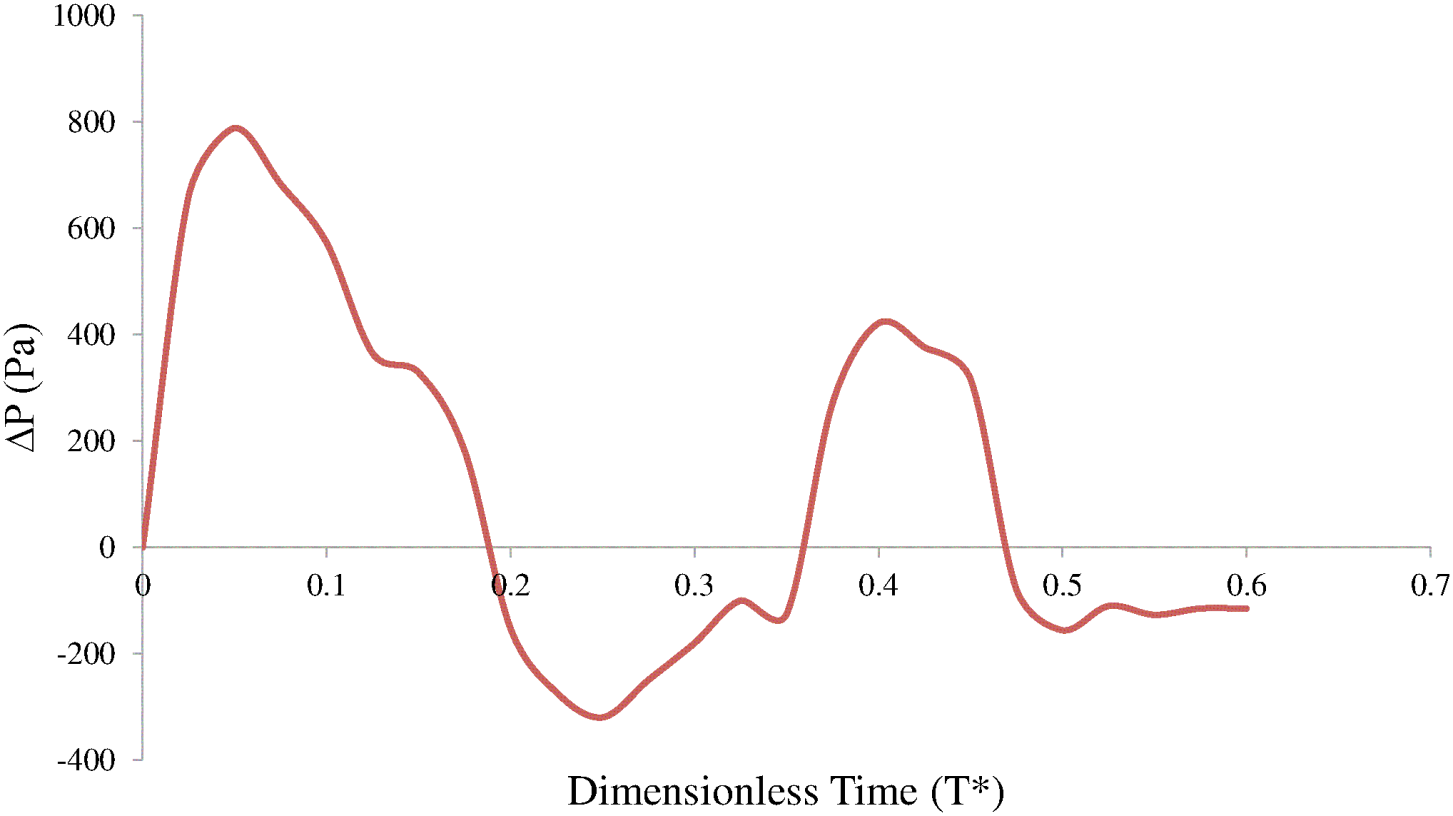
Pressure difference Δ*P*(t*) during diastole.

Fig 6 shows the pressure difference between the instantaneous pressure at the inlet of atrium and that at apex during diastole, which is indicated by Δ*P*(T*) = *P*_*inlet*_−*P*_*apex*_. The pressure difference is related to the acceleration and deceleration of transvalvular jet flow. As expected, the maximum pressure difference occurred during the rapid filling phase and opened the mitral valve rapidly. During the diastasis, the pressure difference was negative and slowed down the transvalvular flow. The other peak of pressure difference was related to the atrial systole, however, its magnitude was much smaller. The maximum and minimum pressure differences in rapid filling were 788 Pa and -249 Pa, respectively. The maximum and minimum pressure differences in the atrial systole were 421 Pa and -156 Pa, respectively.

## Discussion

A number of numerical studies have been conducted to demonstrate intraventricular flow in a healthy subject, while the relevant study on the intraventricular flow after implantation of mitral valve is very limited [22,32–34]. Furthermore, the numerical modeling of BMHV mainly focus on aortic position rather than mitral position. In this study, we simulated the development of intraventricular flow during diastole for a BMHV placed at the mitral orifice. In mimicing a healthy subject, the diastolic phase included early rapid filling, diastasis and late atrial filling stages, and the derived velocity waveform had two crests. It was the first attempt to simulate the BMHV at the mitral position to the best of our knowledge, and we found that its behavior was similar to that at the aortic position (Fig 2). It is worth noting that the mean velocity through the mitral valve is lower than that through the aortic valve, leading to lower maximum angular velocity of mitral valve. When the 25 mm BMHV was placed at mitral position, the maximum angular velocity was 30 rad/s occurring at T* = 0.022, while at aortic position, the peak leaflet angular velocity was 37.5 rad/s occurring at T* = 0.019 [26]. The BMHV is symmetric, but these two leaflets behaved slightly differently from each other, due to the non-uniform downstream flow field (ventricular flow). The posterior leaflet hit the blocking mechanism prior to the anterior leaflet. The impact velocity of the posterior leaflet (2.3 rad/s) was lower than that of the anterior leaflet (3.5 rad/s), and these values are comparable to that of an aortic BMHV (3.12 rad/s) [26]. After reaching the maximum opening angle, both leaflets, for the most part, remained in fully open position. This implies that simulating the left ventricle with the leaflets fixed at the maximal position is a feasible option to closely investigate influences of a BMHV on the intraventricular flow structure, since it mitigates the difficulties of handling rotating leaflets [35].

Despite the differences in terms of BMHV type and ventricular geometry employed, the flow pattern predicted in this study basically matches the experimental studies in [18,36]. The jet flow through the leaflet was directed towards posterior rather than anterior direction, and the vortices rotating in the clockwise direction was more intense in terms of vorticity contours. Furthermore, the streamlines indicated a large clockwise vortex dominating the intraventricular flow. As mentioned in [18], the intraventricular flow field obtained from *in-vitro* measurement is an approximation of the physiological condition when no mitral valve is placed at mitral orifice in test rig, i.e. the uniform flow through the mitral orifice. Similarly to the valveless model [18,22,37], the large anterior vortex was initiated by the area expansion near aortic root during the rapid filling phase when BMHV was placed at mitral position in this study and [38]. In contrast, the vortices developed from shear layers on leaflets had less significant impact on intraventricular flow [18,36,39,15]. Normally, a vortex ring is present during the rapid filling phase, while a characteristic triple-jet flow pattern that consisted of two lateral jets and a relatively weak central jet is observed in BMHV [5,40]. As a result, the maximum velocity magnitude through the BMHV is higher. In this model, the mitral BMHV was placed perpendicular to the axis of spheroid and the space between transvalvular flow and posterior wall was limited. As a result, the posterior vortex was significantly constrained and dissipated its energy rapidly. Therefore, the intraventricular flow at the downstream of BMHV was similar to that in a normal subject. In the literature, the posterior vortex varied significantly in size and strength, due to the direction of transvalvular flow and the space preserved form posterior vortex (between mitral orifice and posterior wall). In some studies, posterior vortex exceeded anterior vortex, leading to a counterclockwise ventricular flow [15,41]. As the clockwise intraventricular flow pattern is beneficial to preserving flow momentum and redirecting flow towards aorta [22,37,42], the direction of implanted BMHV plays a role in ventricular efficiency and it is worth further analyses.

Probably due to the difficulties in measuring the intraventricular pressure mapping, it was mentioned in very limited studies. A conspicuous feature of the intraventricular pressure was the core region of anterior vortex. The presence of the low pressure region slightly influenced the opening behaviors of both leaflets, because it affected the pressure gradient around the leaflet tips along the horizontal direction (anterior to posterior wall). Firstly, it suppressed the fully opening of anterior leaflet, leading to asymmetric opening of BMHV when it was near the root of aorta. Secondly, it swung the posterior leaflet, because it made the jet flow through the mitral orifice slanting when it moved towards the apex. The low pressure region moved to the center of the ventricle and did not proceed further downwards during the diastasis. Although another low pressure region was observed at the onset of atrial filling phase, it vanished more rapidly, owing the weaker transvalvular flow. Although the normal left ventricle modelled in [43] did not consider mitral valve, the pressure variations described above were also observed. The pressure difference between atrium inlet and apex, Δ*P*(T*), clearly indicated the flow acceleration and deceleration in the rapid filling and the atrial systole and the same trend in the normal ventricle studied in [43,44]. The high pressure difference in the rapid filling phase was the primary driving force for the inject of blood into ventricle and the rapid opening of mitral leaflets. After the peak of rapid filling, the pressure gradient across the BMHV declined as the flow rate through the mitral orifice decreased. During the diastasis, the pressure near the apex was higher than that near mitral orifice, and resulted in the marginal flow rate through mitral orifice. The pressure gradient variations during atrial systole were similar to those in the rapid filling phase, however, the magnitude of pressure difference was lower, leading to a more uniform pressure distribution. The maximum pressure difference in this study was 788 Pa, whereas that in normal subject was 552 Pa [45]. Therefore, the BMHV induced higher pressure drop than the native mitral valve.

### Limitations

There are a number of limitations in this study. Firstly, the atrial geometry was simplified as a straight tube, leading to a uniform inlet flow, whereas the atrial flow is complex in a normal subject. To some extent, atrial flow could influence leaflet dynamics. However, this is a common assumption in the literature as detailed flow information is usually unavailable [22,33,34]. Secondly, the flow was assumed to be laminar, though peak Reynolds number was about 4000. This was due to the difficulty of generating high resolution boundary grids required to enable Shear-Stress Transport *k*−*ω* turbulence model that is widely used to model low Reynolds number flows [46,47]. In terms of viscous model, simulations of BMHV involving FSI can basically be categorized as laminar model and direct numerical simulation (DNS). The former approach neglects turbulence and the mesh deformation is solved using the ALE approach provided by the CFD solver FLUENT in [7–9,23,26,27], which was adopted in the current study. The DNS approach adopts the fixed grid method with a much higher grid number in the order of 10^7^, and the simulations were conducted using in-house code [1,3,12,31,48–50]. However, it is worth noting that the numerical validation of DNS was conducted under laminar flow at *Re = 100* rather than turbulent flow when simulating vortex-induced vibration of six elastically mounted 2D cylinders [12]. Despite the differences between two approaches, they both have been experimentally validated [29,51]. Thirdly, this study focused only on the simulation of BMHV at the mitral position without experimental validation, however, a number of numerical studies of mechanical heart valve at the aortic position using CFD solver FLUENT have been validated based on the comparisons between numerical and experimental results of angular leaflet motion and planar flow field [6,8,29]. Lastly, the ventricle was idealized as a truncated spheroid, however, in the future, the current framework could be applied to the studies of patient-specific heart model reconstructed from magnetic resonance imaging [52,53].

## Conclusion

In this study, we conducted a numerical simulation of intraventricular flow with implantation of BMHV at the mitral position using FSI approach. The numerical modeling has revealed following features of leaflet dynamics and intraventricular flow. Firstly, in addition to a characteristic triple-jet flow, the BMHV leaflets at mitral position behaved similarly to those at aortic position. Secondly, the beneficial clockwise anterior vortex appeared during diastole when BMHV was deployed perpendicularly to left ventricular long axis. Finally, the development of this framework enables patient-specific numerical investigations of intraventricular flow with a prosthetic mitral valve and will have significant impact on the pre-surgery setting.

## Supporting Information

S1 Text Appendix. (DOC)

S1 Fig 3D mesh generated from ICEMCFD ANSYS. (TIF)

S2 Fig Flowchart of FSI coupling between the blood flow and leaflets. (TIF)

S1 Video Development of out-of-plane vorticity contours during diastole. (AVI)
