# Supplementary material for: Numerical Modeling of Intraventricular Flow during Diastole after Implantation of BMHV
Source: PLoS One. 2015 May 11;10(5):e0126315. doi: 10.1371/journal.pone.0126315 (PMC4427484; doi:10.1371/journal.pone.0126315)
Supplement: S1 Text — (DOC) [file pone.0126315.s001.doc]

# Appendix

As mentioned in [9], the surface of a spheroid with its center located at the origin is expressed by:

$$\frac{x^{2}}{c^{2}}+\frac{y^{2}}{a^{2}}+\frac{z^{2}}{a^{2}}=1$$

The parameters $a$ and $c$indicate the short and long axis of the spheroid, respectively. To truncate the spheroid, the position of the intersection between a cylinder (with diameter of $2r$, $r\leq a$) and the spheroid is calculated

$$\begin{matrix} \frac{x^{2}}{c^{2}}+\frac{y^{2}}{a^{2}}+\frac{z^{2}}{a^{2}}=1 \\ y^{2}+z^{2}=r^{2} \end{matrix}$$

which can be solved as

$$x^{2}=\left(1-\frac{r^{2}}{a^{2}}\right)\cdot c^{2}$$

resulting in

$$x=\pm c\sqrt{1-\frac{r^{2}}{a^{2}}}$$

The volume of the truncated spheroid is calculated as

$$\begin{matrix} \int_{x=-c\sqrt{1-\frac{r^{2}}{a^{2}}}}^{c} \int_{y=-a\sqrt{1-\frac{x^{2}}{c^{2}}}}^{a\sqrt{1-\frac{x^{2}}{c^{2}}}} \int_{z=-a\sqrt{1-\frac{x^{2}}{c^{2}}-\frac{y^{2}}{a^{2}}}}^{a\sqrt{1-\frac{x^{2}}{c^{2}}-\frac{y^{2}}{a^{2}}}} dzdydx \\ =\pi a^{2}c\left(\frac{2}{3}+\sqrt{1-\frac{r^{2}}{a^{2}}}+\frac{\left(-\sqrt{1-\frac{r^{2}}{a^{2}}}\right)^{3}}{3}\right) \end{matrix}$$

The parameters $a$ and $c$, which determine the shape of the spheroid, are a function of time. Therefore, at every time level, $t$ , the volume $V(t)$ is given by

$$V(t)=\pi\cdot a(t)^{2}\cdot c(t)\left(\frac{2}{3}+\sqrt{1-\frac{r^{2}}{a(t)^{2}}}+\frac{\left(-\sqrt{1-\frac{r^{2}}{a(t)^{2}}}\right)^{3}}{3}\right)$$

Moreover, the coefficient $a(t)$ is set as a constant relation with $c(t)$

$$a(t)=\frac{c(t)}{\alpha}$$

The spheroid is controlled by one parameter $c(t)$, because the parameter $\alpha$is fixed. Finally, it becomes

$$V(t)=\pi\cdot\frac{c(t)^{3}}{\alpha^{2}}\left(\frac{2}{3}+\sqrt{1-\frac{\alpha^{2}\cdot r^{2}}{c(t)^{2}}}+\frac{\left(-\sqrt{1-\frac{\alpha^{2}\cdot r^{2}}{c(t)^{2}}}\right)^{3}}{3}\right)$$

Figure S1 shows the mesh generated by ICEMCFD ANSYS (Version 14.0) and a half of the casing of BMHV is blanked for the ease of illustrating the leaflet. As demonstrated in this figure, dense grids were generated around the leaflet in order to predict its angular motion accurately.

**Figure S1: 3D mesh generated from ICEMCFD ANSYS**

As demonstrated in Figure S2, the numerical simulation consists of time advancement cycle and an FSI cycle, and Scheme script and UDFs are the additional tools. The Scheme script controlled the overall process of simulation, and UDFs were used to represent rigid leaflets. At $t=0s$, all variables were initialized and the cas and dat files were saved. To modeling the flow at $t=(n+1)\Delta t$, the cas and dat files at $t=n\Delta t$ were loaded into FLUENT firstly. During the first FSI sub-iteration for $k=0$, the angular acceleration was assumed to be the same as that at the previous time step as an initial guess. The angular acceleration at the following FSI sub-iteration was calculated by Eq. 5. Consequently, the current angular position was updated according to the current angular acceleration and previous angular position. The grids were updated automatically in FLUENT and the flow field was modeled after solving Navier-Stokes equations. UDFs were then activated to calculate the torque for convergence check. The relative convergence criterion was a residual drop of three orders of magnitude, and the absolute threshold was $10^{-12}Nm$. If convergence criteria were satisfied, the cas and dat files at current time step were saved and the simulation proceeded to next time step. Otherwise, another FSI sub-iteration was activated. The simulation continued until $T_{end}$was reached. More detailed information is available in [27].

**Figure S2: Flowchart of FSI coupling between the blood flow and leaflets.**
