# Supplementary figures and images for: Numerical Modeling of Intraventricular Flow during Diastole after Implantation of BMHV
Source: PLoS One. 2015 May 11;10(5):e0126315. doi: 10.1371/journal.pone.0126315 (PMC4427484; doi:10.1371/journal.pone.0126315)

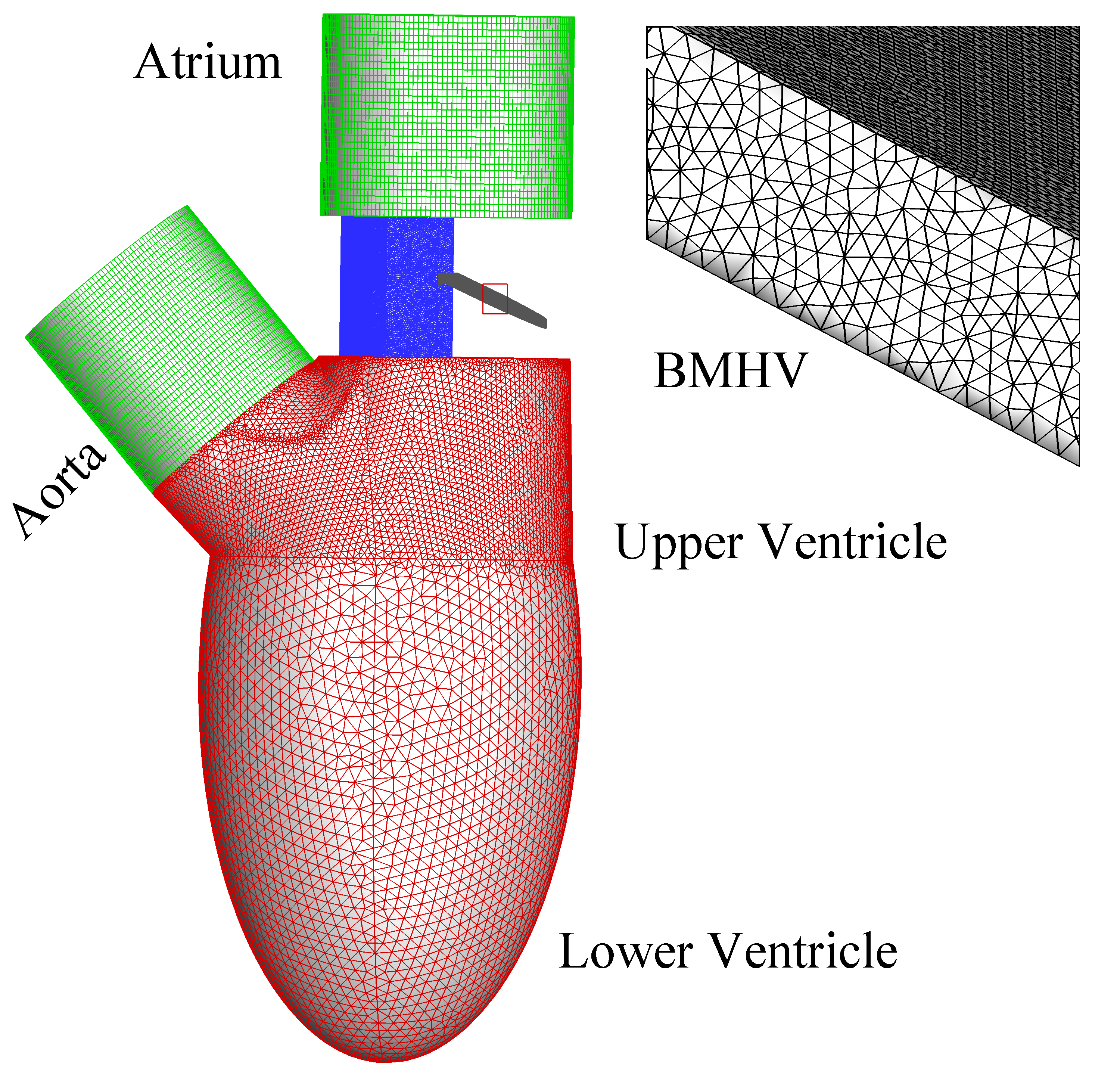

Supplement: S1 Fig — (TIF) [file pone.0126315.s002.tif]

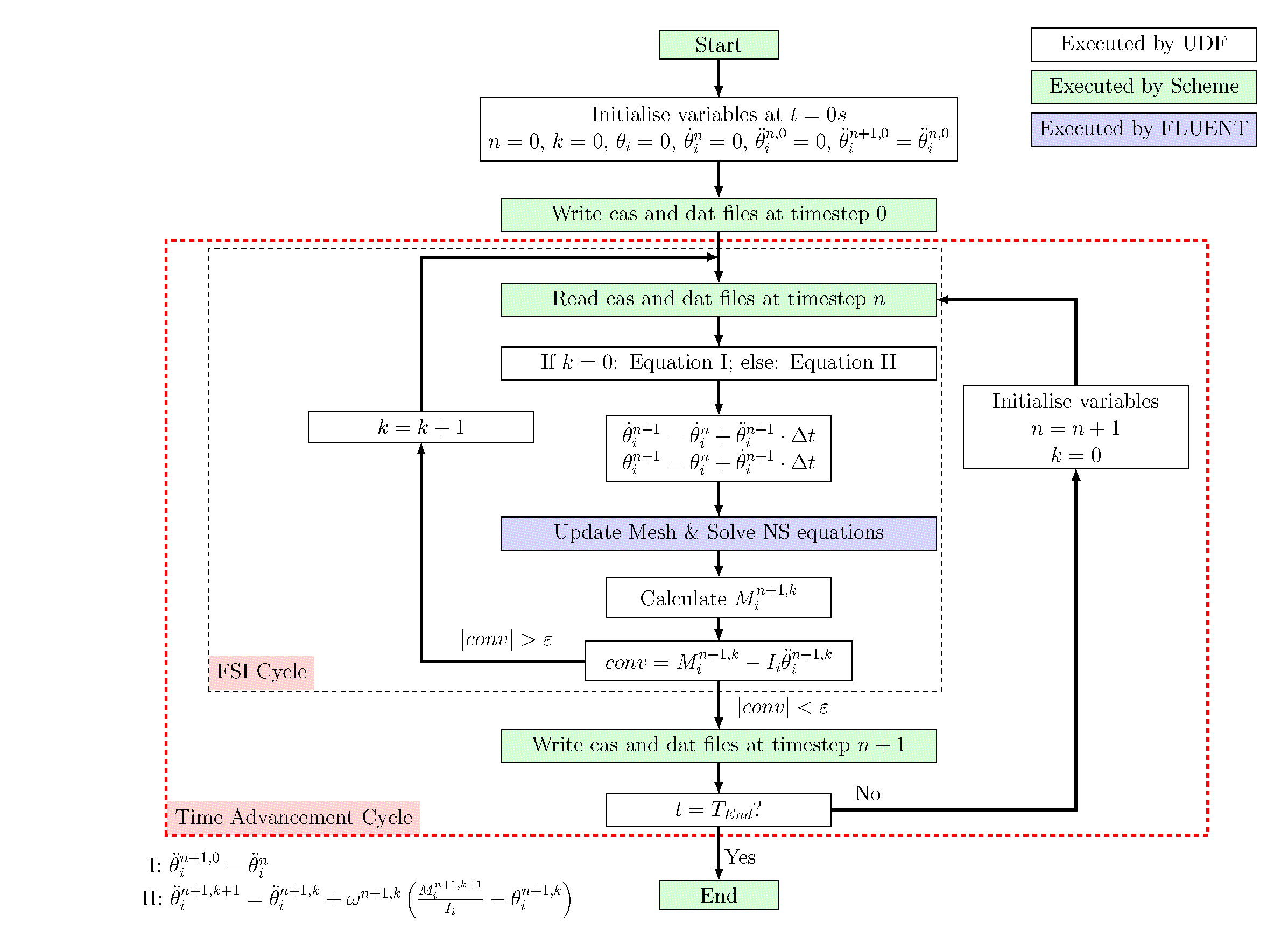

Supplement: S2 Fig — (TIF) [file pone.0126315.s003.tif]
